# Circulating circular RNA profiles associated with celiac disease seropositivity in children with type 1 diabetes

**DOI:** 10.3389/fped.2022.960825

**Published:** 2022-09-23

**Authors:** Juan-juan Zhang, Jun-qi Wang, Xu Xu, Li-dan Zhang, Cai-ping Zhang, Wen-li Lu, Wei-qiong Gu, Zhi-ya Dong, Yuan Xiao, Zhen-wei Xia

**Affiliations:** ^1^Department of Pediatrics, Ruijin Hospital, Shanghai Jiao-Tong University School of Medicine, Shanghai, China; ^2^Department of Endocrinology and Metabolism, Ruijin Hospital, Shanghai Jiao-Tong University School of Medicine, Shanghai, China

**Keywords:** type 1 diabetes, celiac disease autoantibodies, islet autoantibodies, cellular immunity, human leukocyte antigen, circular RNAs

## Abstract

**Introduction:**

The frequency of celiac disease autoantibody (CDAb) positivity in type 1 diabetes (T1D) has increased due to unclear mechanisms, including autoimmune injury. Circular ribonucleic acids (circRNAs) participate in autoimmune diseases, but the roles of circRNAs in T1D with CDAbs are currently unknown. This study aimed to determine the frequency of CDAbs in Chinese children with T1D and describe the relationship between CDAbs and circRNAs.

**Materials and methods:**

Eighty patients diagnosed with T1D were screened for CDAbs and CD-predisposing genes, and circRNAs in peripheral blood mononuclear cells (PBMCs) were collected from 47 patients. The Gene Expression Omnibus (GEO) database was searched for candidate circRNAs in related studies on T1D PBMCs. Data on clinical characteristics (i.e., blood glucose control, residual islet function, and daily insulin dosage) and immunophenotypes (i.e., islet autoantibodies and immune cell subsets) were collected.

**Results:**

In total, 35.0% of patients were positive for CDAbs. CD-predisposing genes accounted for 52.5% of the genes, and no significant difference in frequency was found between the CDAb-positive (CDAb^+^) and CDAb-negative (CDAb^–^) groups. In addition, among the differentially expressed circRNAs from the GEO database, five highly conserved circRNAs homologous to humans and mice were screened, and only the expression of *hsa_circ_0004564* in the CDAb^+^ group significantly decreased (CDAb^+^ vs. CDAb^–^:1.72 ± 1.92 vs. 11.12 ± 8.59, *p* = 6.0 × 10^–6^), while the expression of *hsa_circ_0004564* was upregulated in the general T1D population. Moreover, its parental gene *RAPH1* was significantly upregulated (CDAb^+^ vs. CDAb^–^:1.26 ± 0.99 vs. 0.61 ± 0.46, *p* = 0.011). Importantly, the positive correlation between *hsa_circ_0004564* and CD3^+^ cells was validated in children with T1D after adjustments for CDAbs (*p* = 0.029), while there were no correlations between *hsa_circ_0004564* and clinical characteristics or other immune cell subsets (i.e., CD4^+^ T cells, CD8^+^ T cells, and natural killer cells).

**Conclusion:**

This study highlights the importance of screening for CD in Chinese children with T1D, considering the high prevalence of CDAb positivity and CD-predisposing genes. The profile of candidate circRNAs in children with T1D with CDAbs was different from that in previous reports on general T1D patients from the GEO database. Moreover, *hsa_circ_0004564* and its parental gene *RAPH1* may be new targets for studying immune mechanisms in children with T1D and CD.

## Introduction

Type 1 diabetes (T1D) and celiac disease (CD) are the two most common autoimmune diseases in Western countries. The estimated incidence of T1D continues to increase by 1.4% annually, and the prevalence of CD is estimated to be 1.4% worldwide. CD occurs in 3–16% of patients previously diagnosed with T1D (1). It is an autoimmune enteropathy caused by permanent susceptibility to gluten in genetically susceptible individuals (*human leukocyte antigen* [*HLA*]-*DQ2/DQ8*) and causes atrophy of the gastrointestinal mucosa and malabsorption syndrome (2). Currently, because of the high (>90%) similarity of the haplotype *HLA-DQ2/DQ8* between T1D and CD, the European Society for Pediatric Gastroenterology, Hepatology, and Nutrition (ESPGHAN), American Diabetes Association, and American College of Gastroenterology clinical guidelines recommend the use of CD autoantibodies (CDAbs) as markers to identify enteropathy in patients with T1D and CD (3).

The co-occurrence of CD and T1D is associated with numerous complications that can lead to periods of hypoglycemia and poor diabetes control (2). In patients with T1D, the coexistence of CD affects the intestinal absorption of nutrients and skeletal metabolism and possibly contributes to a higher risk of cardiovascular incidents, even exaggerating complications, such as nephropathy, retinopathy, and neuropathy. A CD diagnosis of ≥ 15 years has shown to be associated with a 2.8-fold increased risk of death in individuals with T1D (4). In China, only two studies on CD in patients with T1D were published in 2016 and 2017 (5, 6). There are insufficient data on the population of children. Moreover, the pathological mechanisms underlying the coexistence of CDAb and T1D remain largely unknown.

Circular ribonucleic acids (circRNAs) are covalently linked single-stranded RNAs without 5’ and 3’ ends (7), with the majority of them having the following characteristics: they are abundant, conserved, stable, and specific (8). Dysregulation of circRNAs contributes to the pathogenesis of autoimmune diseases (9), and may serve as potential biomarkers and immune regulators, offering potential opportunities for the identification of new therapies (10). Previous findings have corroborated that circRNAs are involved in proinflammatory cytokine-mediated β-cell dysfunction and suggest their involvement in the development of T1D (11, 12).

## Materials and methods

Eighty patients diagnosed with T1D at the Department of Pediatrics, Ruijin Hospital, affiliated with Shanghai Jiao-Tong University School of Medicine, Shanghai, China, from January 2017 to December 2021 were enrolled. All of them were of the Han Chinese ethnicity. T1D was diagnosed in accordance with the International Society for Pediatric and Adolescent Diabetes criteria (13): acute-onset ketosis or diabetic ketoacidosis (DKA), insulin therapy, and a previous diagnosis of T1D autoantibodies. Patients with IgA deficiency and those receiving immunosuppressive treatment were excluded from the study. The studies involving human participants were reviewed and approved by the Ethics Committee of Ruijin Hospital and performed in accordance with the Declaration of Helsinki, 2000. The participants provided their written informed consent to participate in this study.

### Clinical and laboratory data

On the day of diagnosis, blood samples were obtained and centrifuged to collect serum. Demographic data were collected from patient files, including data on height standard deviation score (SDS), weight SDS, body mass index (BMI) SDS, frequent hypoglycemia (more than three times per week), abdominal pain, chronic diarrhea, and constipation. All patients were followed up with regard to age at baseline, glycated hemoglobin [HbA1c, measured using high-pressure liquid chromatography], residual islet function (fasting C-peptide [FCP] level, stimulated C-peptide [PCP] level during a mixed meal tolerance test [Roche Diagnostics, Penzberg, Germany]), and daily insulin dosage (DID) (IU/kg/d). Humoral immunity profiles were tested using islet autoantibodies, including glutamic acid decarboxylase 65 antibody (GAD), insulin autoantibody (IAA), and islet cell antigen antibody (ICA). Serum levels of GAD were measured using a commercial radioimmunoassay kit (RSR Limited, Cardiff, Wales, UK), and the results were considered positive when the values were >7.5 units/mL. IAA and ICA positivity was determined by enzyme-linked immunosorbent assay (ELISA; RSR Limited, Cardiff, Wales, UK).

### Analysis of immune cell subsets

Peripheral blood mononuclear cells (PBMCs) from 80 patients with T1D were isolated. Forward and side scatter measurements were combined with CD45 to identify lymphocytes and exclude monocytes. The absolute subpopulations of lymphocyte numbers were calculated based on the total lymphocyte counts and the percentage of lymphocyte cell subpopulations, as identified by flow cytometry using EPICS XL (Beckman Coulter, Brea, CA, USA) and Divasoftware (BD Biosciences, Franklin Lakes, NJ, USA). The fraction of lymphocyte cell subsets was determined by multicolor fluorescence-activated cell sorter analysis using appropriate surface markers (Beckman Coulter): anti-CD3-FITC (Clone UCHT1), anti-CD4-PE-Cy7 (Clone 13B8.2), anti-CD8-APC-H7 (Clone B9.11), anti-CD16-PE (Clone 3GB), and anti-CD56-PE (Clone N901).

### Screening for celiac disease autoantibodies and celiac disease-predisposing genes

Participants were screened for the presence of human anti-endomysial antibody (EMA; EMA-IgA or IgG, FA1914-1010A/FA1914-1010G, indirect immunofluorescence), human tTG antibodies (tTG-IgA or IgG, EV1910-9601A/EV 1910-9601G), and human deaminated gliadin peptide (DGP) antibody (DGP-IgA or IgG, EV3011-9601A/EV3011-9601G) using an ELISA kit (Oumeng, China). A tTG-IgA level ≥20 RU/mL, a tTG-IgG level ≥25 RU/mL, the presence of EMA-IgA/IgG, and a DGP-IgA/DGP-IgG level ≥25 RU/mL were considered positive for CD. The inter-assay coefficient variations were <7.2, <9.2, <8.9, and <11.4% for the tTG-IgA, tTG-IgG, DGP-IgA, and DGP-IgG levels, respectively.

The presence of haplotypes associated with a predisposition to CD was evaluated in all patients. Genomic DNA was isolated from peripheral leukocytes using the QiaAMP^®^ DNA mini kits (Qiagen GmbH, Hilden, Germany). The haplotypes for *HLA-DQ2* (*HLA-DQ2.2* [DQA1 * 02:01, DQB1 * 02:01/02:02] or *HLA-DQ2.5* [DQA1 * 05:01/05:05, DQB1 * 02:01/02:02]) and *HLA-DQ8* (DQA1 * 03:01/03:02/03:03, DQB1 * 03:02) were analyzed using microarray method (MN 5310-2005, Oumeng, China).

### Bioinformatics analysis

The Gene Expression Omnibus (GEO) database was searched for related studies on T1D PBMC, and finally GSE133225 (circRNA and mRNA expression microarray data) was selected as our study. GSE133225 was uploaded to the GEO database by Caiyan Zhang of Fudan Pediatric Hospital in 2019. In this study, PBMCs from patients with T1D were used as samples for gene chips. GSE133225 was based on GPL22120 Agilent-078298 human ceRNA array V1.0 4 × 180K [Probe Name Version] chip platform to detect samples of four patients with T1D and four healthy people. After the gene chip had passed quality control and rejected unqualified samples, the data underwent preprocessing steps such as background correction, data standardization, and summarization to obtain the gene expression matrix required for further analysis. The expression matrix was then subjected to principal component analysis (PCA) with cluster analysis and limma to calculate the differentially expressed genes (| log (fold change)| >1 and *p* < 0.05).

Gene Ontology (GO) and Kyoto Encyclopedia of Genes and Genomes (KEGG) Pathway Analysis: The predicted circRNAs were selected for GO term and KEGG pathway analysis using the Database for Annotation, Visualization, and Integrated Discovery (DAVID). The -log10 (*p* value) indicates the enrichment score, which represents the significance of the GO term and KEGG pathway enrichment.

### RNA extraction and reverse transcription quantitative PCR

Peripheral blood mononuclear cells were isolated from the peripheral blood of 47 of 80 patients. Total RNA was extracted using TRIzol (Thermo Fisher Scientific, USA), followed by purification with the QIAamp RNA Blood Mini Kit (Qiagen GmbH, Hilden, Germany), and eluted in 20 μL of diethyl pyrocarbonate-treated water. cDNA was synthesized from equal quantities of total RNA (1.5 μg) in a 20-μL reverse transcription reaction using the PrimeScript™ RT Master Mix RR036B (Takara, Japan) according to the manufacturer’s instructions. Real-time quantitative PCR was performed with TB Green^®^ Premix Ex Taq™ II RR820B (Takara, Japan) using the QuantStudio 6 Real-Time PCR System (Thermo Fisher Scientific, USA). β*-actin* (Sangon, Shanghai, China) was used as an internal control, and the fold change was calculated using the 2^–ΔΔ*Ct*^ method. The primer pairs for circRNAs (RiboBio, Guangzhou, China) and the parent gene (Sangon, Shanghai, China) used for qPCR are listed in Supplementary Table 1.

### Statistical analysis

The results are shown as mean ± standard deviation or median (interquartile range) or documented as positive cases, constituent ratios, or ratios. We compared different CDAb groups using an independent sample *t*-test for continuous data and the chi-square test for binary data. ANOVA was conducted among the groups, with a model that included demographics (age, sex, and duration of diabetes) as covariates. The relationship between circRNAs and clinical data was tested using Pearson’s correlation and linear regression. When continuous data were not normally distributed, a non-parametric test was used for comparisons. Statistical significance was set at *p* < 0.05. Statistical analyses were performed using SPSS (version 17.0; SPSS Inc., Chicago, IL, USA).

## Results

### Celiac disease autoantibodies in the children with type 1 diabetes

**FIGURE 1:**
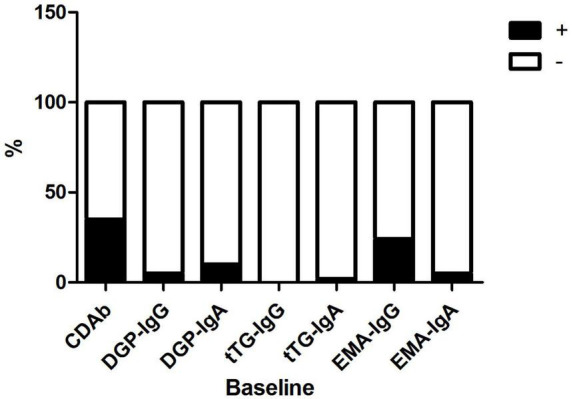
Distribution of CDAbs in children with T1D. CDAbs, celiac disease autoantibodies; T1D, type 1 diabetes.

The mean patient age was 8.9 ± 3.7 years; 41 (51.3%) patients were male and 39 (48.7%) were female. The median duration of diabetes was 2.5 (1.0, 6.7) months. Of the 80 patients, 28 (35.0%) were seropositive for CDAbs (EMA-IgA or IgG, tTG-IgA or IgG, DGP-IgA or IgG, or both). The incidence of the presence of these CDAbs was quite different. EMA-IgG positivity was detected in 19 of 80 (23.8%) patients. Both DGP-IgG and EMA-IgA positivity were detected in four (5%) and eight (10.0%) children, respectively. tTG-IgA positivity was a rare occurrence, appearing in only two (2.5%) children. None of the children tested positive for tTG IgG (Figure 1). Only one boy was found to have three CDAbs (tTG-IgA, EMA-IgG, and DGP-IgA), and seven patients were found to have two CDAbs (three for EMA-IgG and EMA-IgA [two girls and one boy], two for DGP-IgG and EMA-IgG [one girl and one boy], one for DGP-IgA and DGP-IgG, and one for DGP-IgA and EMA-IgG). There were no significant differences in the clinical characteristics and immunophenotypes between the CDAb^+^ and CDAb^–^ groups (Table 1).

**TABLE 1:** Comparison of the anthropometric features of children with T1D possessing positive or negative celiac serologies.

	CDAb^+^ (*n* = 28)	CDAb^–^ (*n* = 52)	*p*-value
Age at baseline (y)	8.42 ± 4.36	9.22 ± 3.36	0.36^†^
Sex (M/F)	13/15	28/24	0.53^¶^
Duration of diabetes (mo)	6 (1.00, 7.75)	2 (1.00, 6.00)	0.27*
Height SDS	0.36 (−0.30,1.32)	0.48 (−0.33,1.19)	0.91*
Weight SDS	0.24 (−0.31,0.72)	0.11 (−0.33,0.63)	0.52*
BMI SDS	−0.04 (−0.57,0.68)	−0.12 (−1.00,1.43)	0.55*
Frequent hypoglycemia (%)	5 (17.9)	10 (19.2)	0.88^¶^
Abdominal pain (%)	1 (3.6)	3 (5.8)	1.00^¶^
Abnormal stool (%)	2 (7.1)	2 (3.8)	0.61^¶^
FCP (ng/mL)	0.43 (0.24, 0.89)	0.49 (0.23, 0.82)	0.81*
PCP (ng/mL)	0.86 (0.28, 1.33)	0.84 (0.51, 1.48)	0.47*
HbA1c (%)	9.14 ± 2.77	10.38 ± 2.80	0.63^#^
DID (IU/kg/d)	0.69 ± 0.32	0.71 ± 0.28	0.17^#^
CD3^+^ cell (%)	69.54 ± 6.80	74.21 ± 5.29	0.09^#^
CD4^+^ T cell (%)	39.86 ± 7.22	40.42 ± 6.94	0.93^#^
CD8^+^ T cell (%)	23.41 ± 5.59	27.15 ± 7.06	0.12^#^
CD4/CD8 (%)	1.83 ± 0.68	1.61 ± 0.57	0.37^#^
NK (%)	8.32 ± 5.94	7.97 ± 4.14	0.70^#^

*Mann–Whitney U test. ^†^Student’s t-test. ^¶^χ^2^ test, or Fisher’s exact test. ^#^ANOVA after adjusting for age, sex, and duration of diabetes. Data are presented as mean ± standard deviation or median (interquartile range: 25th–75th percentile). SDS, standard deviation score; BMI, body mass index; HbA1c, glycated hemoglobin; FCP, fasting C-peptide; PCP, stimulated C-peptide; DID, daily insulin dosage; NK, natural killer.

### Profiles of the celiac disease-predisposing genes in children with type 1 diabetes possessing celiac disease autoantibodies

**FIGURE 2:**
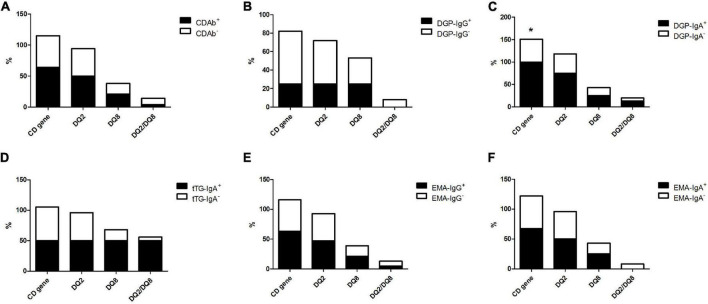
Profile of the CD-predisposing genes in children with T1D with CDAbs. (*p < 0.05, **p < 0.01, ***p < 0.001). **(A)** CDAbs. **(B)** DGP-IgG. **(C)** DGP-IgA. **(D)** tTG-IgA. **(E)** EMA-IgG. **(F)** EMA-IgA. Data are expressed as percentages. χ^2^ or Fisher’s exact test. CD, celiac disease; CDAbs, celiac disease autoantibodies; T1D, type 1 diabetes; DGP, deaminated gliadin peptide; Ig, immunoglobulin; tTG, tissue transglutaminase; EMA, endomysial antibody.

Of all patients with T1D, 52.5% had CD-predisposing genes, and the most common haplotype was *DQ2* (46.3%), followed by *DQ8* (18.8%), and *DQ2/DQ8* (7.5%). In the CDAb^+^ group, 16 (64%) children with T1D had CD predisposing genes. *DQ2* (50.0%) was the most common haplotype, followed by *DQ8* (21.4%) and *DQ2/DQ8* (3.6%). Meanwhile, in the CDAb^–^ group, the most common haplotype was *DQ2* (44.2%), followed by *DQ8* (17.3%) and *DQ2/DQ8* (9.6%). No correlation was found between CDAbs and CD-predisposing genes (Figure 2).

Conversely, the difference in the frequency of CD-predisposing gene occurrence between the DGP-IgA^+^ subgroup (100%) and DGP-IgA^–^ subgroup (50.7%) was significant, whereas no association was found between the other CDAb subgroups and genetic evidence of CD. There was a trend of a higher frequency of CD-predisposing genes in all CDAb^+^ subgroups, except for the DGP-IgG^+^ subgroup, although no significant relationship was found (Figure 2).

### Screening of the candidate circRNAs

**FIGURE 3:**
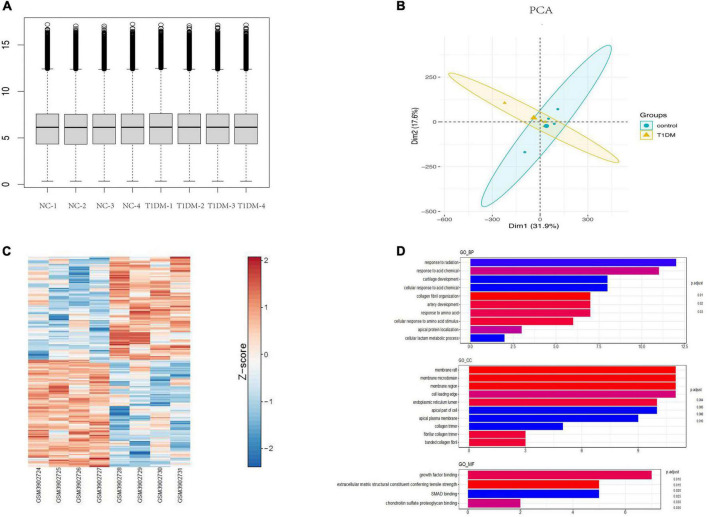
Bioinformatics analysis of circRNAs in T1D. **(A)** Normalization of circRNA data. **(B)** Principal component analysis chart. **(C)** A heat map according to the circRNA expression level ranking. **(D)** GO terms analysis of upregulated and downregulated circRNAs. T1D, type 1 diabetes; GO, Gene Ontology; BP, biological process; CC, cellular component; MF, molecular function.

Using the limma package for data preprocessing, the median and distribution of gene expression between samples in each dataset were found to be similar (Figure 3A). PCA with cluster analysis was performed on the GSE133225 data expression matrix, and the PCA chart showed obvious clustering between the groups (Figure 3B). A heat map was drawn according to the circRNA expression level ranking (Figure 3C) and differential genes were calculated using limma (| log (fold change)| > 1 and *p* < 0.05). Differentially expressed circRNAs were compared between the T1D and healthy control groups, and 78 significantly upregulated and 84 significantly downregulated circRNAs were identified. These analyses revealed that the expression levels of circRNAs were significantly different between T1D patients and healthy controls.

GO analysis of differentially expressed parental genes in GSE133225 showed that incremental genes were mainly enriched in cellular components (CC), including membrane raft, membrane microdomain, and membrane region; in molecular function (MF), including growth factor binding; and in biological processes (BP), including response to radiation and response to acid (Figure 3D). Among the differentially expressed circRNAs, five highly conserved circRNAs homologous to those in humans and mice were screened for qPCR validation (Supplementary Table 2).

### Validation of the candidate circRNAs

For the five candidate circRNAs, six patients with T1D were first selected for the pre-experiment and grouped according to CDAbs to compare the expression differences of *hsa_circ_0006561*, *hsa_circ_0004564*, *hsa_circ_0041267*, *hsa_circ_0004712*, *hsa_circ_0018827* between the groups. The results indicated that compared with the CDAb^–^ group, the expression of *hsa_circ_0004564* in the CDAb^+^ group was significantly decreased (CDAb^–^ vs. CDAb^+^:4.37 ± 2.72 vs. 1.06 ± 0.43, *p* = 0.0487) (Table 2).

**TABLE 2:** Correlation between candidate circRNAs and celiac serology in the children with T1D.

	CDAb^+^ (*n* = 3)	CDAb- (*n* = 3)	*p*-value
Age at baseline (y)	9.00 ± 5.87	9.22 ± 5.87	0.40^†^
Sex (M/F)	3/0	2/1	1.00^¶^
Duration of diabetes (mo)	3.0 (1.5,108)	4.0 (0.5,5)	0.83*
*hsa_circ_0006561*	1.00 ± 0.06	0.99 ± 0.56	0.91^†^
*hsa_circ_0004564*	1.06 ± 0.43	4.37 ± 2.72	0.0487^†^
*hsa_circ_0041267*	1.16 ± 0.65	1.88 ± 0.60	0.9212^†^
*hsa_circ_0004712*	1.09 ± 0.49	0.65 ± 0.08	0.0512^†^
*hsa_circ_0018827*	1.15 ± 0/76	0.87 ± 0.53	0.6491^†^

*Mann–Whitney U test. ^†^Student’s t-test. ^¶^χ^2^ test, or Fisher’s exact test. Data are presented as mean ± standard deviation or median (interquartile range: 25th–75th percentile).

**FIGURE 4:**
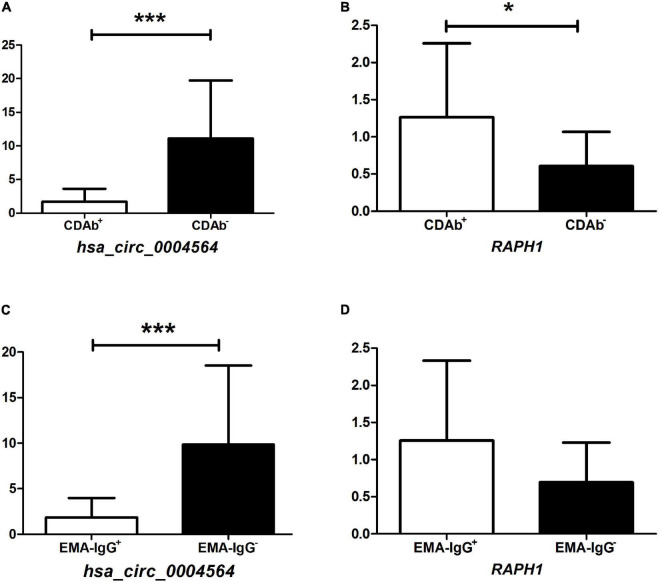
Association of *hsa_circ_0004564* and *RAPH1* in children with T1D with CDAb positivity. (*p < 0.05, ^**^p < 0.01, ^***^p < 0.001). Vertical lines indicate one standard deviation above and below the mean. Student’s *t*-test. **(A)** *hsa_circ_0004564* with CDAbs. **(B)** *RAPH1* with CDAbs. **(C)** *hsa_circ_0004564* with EMA-IgG. **(D)** *RAPH1* with EMA-IgG. CDAbs, celiac disease autoantibodies; T1D, type 1 diabetes; EMA, endomysial antibody; Ig, immunoglobulin.

**FIGURE 5:**
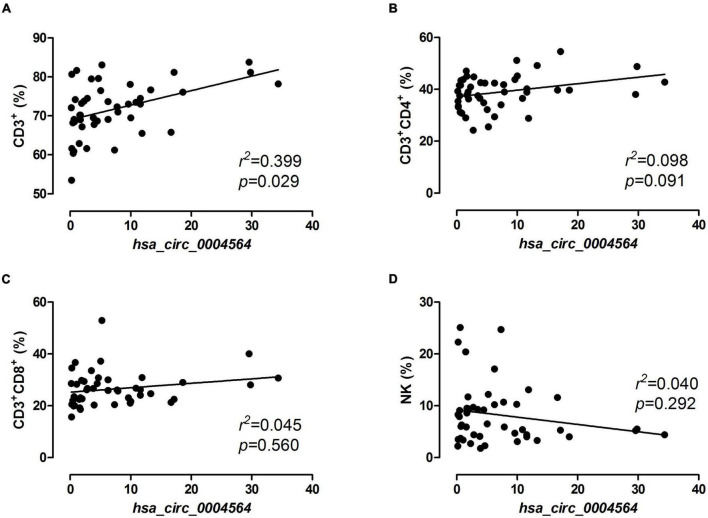
Correlation analysis of *hsa_circ_0004564* expression and immune cell subsets in children with T1D. Pearson’s correlation and linear regression after adjustments for CDAbs, age, sex, and duration of diabetes. **(A)** CD3^+^ cell. **(B)** CD4^+^ T cell. **(C)** CD8^+^ T cell. **(D)** NK cell. CDAbs, celiac disease autoantibodies; T1D, type 1 diabetes; NK, natural killer cells.

By comparing *hsa_circ_0004564* and its parental genes *RAPH1* (*Ras associated [RalGDS/AF-6] and pleckstrin homology domains 1*) in the PBMCs of children with T1D according to the presence of CDAbs (Supplementary Table 3), the results suggested that compared with the CDAb^–^ group, the expression of *hsa_circ_0004564* was significantly decreased in the CDAb^+^ group (CDAb^–^ vs. CDAb^+^:11.12 ± 8.59 vs. 1.72 ± 1.92, *p* = 6.0 × 10^–6^) and the expression of *RAPH1* was significantly upregulated in the CDAb^+^ group (CDAb^–^ vs. CDAb^+^:0.61 ± 0.46 vs. 1.26 ± 0.99, *p* = 0.011) (Figures 4A,B). Importantly, the positive correlation between *hsa_circ_0004564* and CD3^+^ cells was validated in children with T1D after adjustments for CDAbs (*p* = 0.029) (Figure 5A), while no correlations were observed between *hsa_circ_0004564* and HbA1c, FCP, PCP, DID, types of islet autoantibodies, or other immune cell subsets (i.e., CD4^+^ T cells, CD8^+^ T cells, and NK cells) (Figures 5B–D). No correlations were observed between *RAPH1* expression, clinical characteristics, and immunophenotypes.

Subsequently, by further evaluating the correlation between the expression of *hsa_circ_0004564* and its parental gene *RAPH1* in children with T1D between the CDAb subgroups, the expression of *hsa_circ_0004564* in the EMA-IgG subgroup was found to be significantly different. *hsa_circ_0004564* was significantly lower in the EMA-IgG^+^ subgroup than in the EMA-IgG^–^ subgroup (EMA-IgG^+^ vs. EMA-IgG^–^:1.84 ± 2.13 vs. 9.85 ± 8.68, *p* = 2.2 × 10^–5^), although the expression of *RAPH1* was higher in EMA-IgG^+^ subgroup without statistical difference (*p* = 0.063) (Figures 4C,D). No association was found between *hsa_circ_0004564* and *RAPH1* in the other CDAb subgroups.

## Discussion

In this study, the prevalence of CDAb positivity was relatively high among Chinese children with T1D (35.0%); 23.8% of the patients had EMA-IgG, whereas only 2.5% had tTG-IgA. A large discrepancy was also observed between the CDAb tests performed based on data from previous studies. Thirty-one studies, including 63,349 children from Caucasian and Indian populations with T1D, found that the use of CDAb tests was greater than that of intestinal biopsy (1.4–24.5% vs. 1.1–16.6%) (14). In a meta-analysis of 18 studies, the prevalence of CDAbs in the general Chinese population was 0.27% (95% CI, 0.02–0.71). The prevalence of CDAbs in high-risk Chinese populations (including T1D, rheumatoid arthritis, ankylosing spondylitis, psoriasis, inflammatory bowel disease, irritable bowel syndrome, colitis, anemia, chronic diarrhea, abnormal stools, low body mass index, and short stature) was 8.34% (95% CI, 4.90–12.54), which was significantly higher than that in the general population (OR 7.27, 95% CI, 4.06–13.04). The prevalence of biopsy-proven CD in high-risk Chinese populations was 4.44% (95% CI, 1.53–8.58) (15). A study conducted by the Hospital of Jilin University in China between 2010 and 2013 (5) showed that the positivity rate of tTG-IgA in patients with T1D was 22% (39/178). This discrepancy may be related to the differences in regional diet and patient age; the Hospital of Jilin University cohort was from North China and consumed a diet mainly consisting of wheat, while most patients in our research cohort were from East China and consumed a diet mainly consisting of rice. This indicates that the patients in our study had relatively low consumption of gluten protein (16). Furthermore, the patients in the Hospital of Jilin University cohort were almost adults (mean age: 27.2 ± 15.2 years), while the patients in our cohort were children and adolescents (mean age: 8.9 ± 3.7 years). In another study, an age-related difference in tTG-IgA levels was suggested, as the mean tTG-IgA level in the <4.0 years of age group was slightly but significantly lower than that in the ≥4.0 years of age group (17).

The incidence of typical *HLA-DQ2* and *DQ8* haplotypes was very high in patients with CD (90–95% and 5–10%, respectively), and the *DQ2/DQ8* genotype was strongly associated with an increased risk of developing T1D in European populations (18). Current reviews on CD diagnosis have drawn attention to the inclusion of *HLA-DQ* genotyping in addition to serological tests, especially those with a high negative predictive value (19). The frequencies of the occurrence of CD-predisposing *HLA* genes in patients with T1D in our cohort were much higher than those of *HLA-DQ2* (3.4%) and *HLA-DQ8* (2.1%) haplotypes in a general healthy Chinese population (20). Moreover, DGP-IgA positivity was found to be associated with a higher percentage of CD-predisposing *HLA* genes, whereas no relationship was found in other CDAbs, suggesting that there might be different pathogenic pathways according to the occurrence of CDAb types. However, genetic factors alone cannot fully explain the occurrence and development of CD and T1D. Environmental factors have also been implicated in the etiology of T1D and CD. Viral infections and early exposure to gluten or cow’s milk in the infant diet have been implicated in disease pathogenesis. Moreover, breastfeeding, diet, infections, antibiotics, and method of birth alter the composition of the microbiome. Human data indicate subtle differences in the microbiome of children with T1D autoimmunity, while intestinal dysbiosis has clearly been demonstrated in CD (21).

In our study, no relationship was observed between the CDAb levels and clinical characteristics. There are controversial data regarding blood glucose control and its association with T1D and CD comorbidity, since it has been reported that CD may increase glycemic variability, with either frequent hypoglycemia due to malabsorption in Europe and North America/Canada (4) or no difference in HbA1c levels in Asia (22), thereby leading to an underestimation of poor glycemic control (23). The effect of CDAb on β-cell function in T1D patients is currently unclear. In addition, CDAb also has an uncertain role in insulin dosage, as some authors observed a lower need for insulin during the first two years following the diagnosis of CD in T1D children than in non-CD patients (4, 24), while a higher DID was found to be associated with CDAb positivity (25).

Interestingly, among the five candidate circRNAs from the general T1D patients in the GEO database, only the expression of *hsa_circ_0004564* in the CDAb^+^ group was found to be lower than that in the CDAb^–^ group, while the expression of *hsa_circ_0004564* was upregulated in the general T1D population, suggesting that the expression in the CDAb^–^ group was more obviously upregulated. Hence, there are differences in the profile of circRNAs between the CDAb^+^ and CDAb^–^ groups as well as in the general T1D population. Furthermore, although no relationships were observed between the CDAbs and immunophenotype, it is notable to find that *hsa_circ_0004564* was positively correlated with CD3^+^ cells. The contradictory effects of *hsa_circ_0004564* may be explained by the finding that autoimmunity is an outcome of an imbalance between anti-inflammatory/proinflammatory immune cell ratios (26). Moreover, mechanisms of cellular immunity in T1D and CD: The autoimmune process of T1D is mainly mediated by autoreactive CD4^+^ and CD8^+^ T lymphocytes, and the onset of CD is related to activated CD4^+^ T lymphocytes, which secrete high levels of proinflammatory factors (27). Thus, exploration of the role of *hsa_circ_0004564* on different T cell subsets may help identify the molecular mechanisms underlying T1D and CD. The correlation between EMA-IgG and the expression of *hsa_circ_0004564* was consistent with that of total CDAbs, suggesting that EMA-IgG may play a more important role when T1D merges with CD. In the past, the level of tTG-IgA was more important in the serological diagnosis of CD. According to the latest meta-analysis statistics, the sensitivity and specificity of EMA-IgG for the diagnosis of CD in adults are 39.3 and 98.5%, respectively. Since it is not detectable in all compartments, its true role in the diagnosis of CD may be underestimated (28).

In addition, the parental gene *RAPH1* was negatively regulated by *hsa_circ_0004564* and its expression was significantly higher in the CDAb^+^ group, suggesting that *RAPH1* may be associated with a stronger autoimmune response. *RAPH1*, also known as lamellipodin (LPD), is a downstream effector of the Ras pathway and plays an important role in epithelial–mesenchymal transition (EMT) (29). Previous studies have shown that changes in intracellular tight junctions and weakened cell adhesion properties are critical for the induction of EMT; thus, *RAPH1* plays an important role in regulating cell migration and re-epithelialization (30). High *RAPH1* expression is associated with an aggressive breast cancer phenotype and has independent prognostic value (31); *RAPH1* was recently found to be critical for regulating cell proliferation, as it stimulates extracellular matrix (ECM) stiffness-mediated cyclin expression and intracellular stiffening in mouse embryonic fibroblasts, although increased ECM stiffness leads to abnormal cell cycle progression and proliferation (32); high expression of *RAPH1* can be used as a marker of major depressive disorder (MDD) (33). The expression of LPD in regulatory T cells (Treg) of IL-10–null mice (a mouse model of inflammatory bowel disease [IBD]) is higher than that in conventional T cells, and is involved in the physiological integrin activation of Treg cells, suggesting that LPD may have a key role in immune regulation (34). These findings on *RAPH1* suggest that high expression of *RAPH1* may be involved in the occurrence and progression of autoimmune responses in T1D combined with CD.

Our study had some limitations. First, the small sample size may have contributed to the high CDAb positivity in patients with T1D. Second, endoscopic and histopathological examinations via intestinal biopsy were not performed to confirm CD. Moreover, according to the 2012 ESPGHAN standard, in symptomatic children with high concentrations of tTG-IgA, the diagnosis can be established without biopsy, as confirmed by positive EMA and celiac *HLA* genotypes (35). Therefore, it would be better to perform quantitative measurements of CDAbs to make a clinical diagnosis. Third, serological measurements of CDAbs were performed at a single time point. It has been reported that the combined future damages of T1D with CD can exaggerate complications such as nephropathy, retinopathy, as well as other autoimmune disorders in children with T1D. CD may contribute to lower bone density, renal insufficiency, and a lower quality of life in affected children and adults with T1D. CD may also increase the risk of gastrointestinal tumors (36). Thus, further clinical follow-up data of patients with CDAb positivity are needed to confirm the persistence of CDAbs and possible future development of CD.

## Conclusion

This study highlights the importance of screening for CD in Chinese children with T1D, considering the high prevalence of CDAb positivity and CD-predisposing genes. The profile of candidate circRNAs in children with T1D possessing CDAbs was different from that in previous reports on general T1D patients from the GEO database. Moreover, this study is the first to demonstrate a relationship between *hsa_circ_0004564* and its parental gene *RAPH1* in children with T1D possessing CDAbs. The association of *hsa_circ_0004564* with CD and the relationship between *RAPH1* and T1D have not been previously reported. *Hsa_circ_0004564* and *RAPH1* may be new targets for studying immune mechanisms in children with T1D and CD.

## Data availability statement

The original contributions presented in this study are included in the article/Supplementary material, further inquiries can be directed to the corresponding author.

## Ethics statement

The studies involving human participants were reviewed and approved by Ethics Committee of Ruijin Hospital. Written informed consent to participate in this study was provided by the participants’ legal guardian/next of kin.

## Author contributions

J-JZ and J-QW performed the research, and wrote the manuscript. J-JZ and Z-WX designed the research study. XX, L-DZ, C-PZ, and W-LL contributed essential reagents or tools. W-QG, Z-YD, and YX analyzed the data. All authors contributed to the article and approved the submitted version.

## References

[1] Alshiekh S Maziarz M Geraghty DE Larsson HE Agardh D. High-resolution genotyping indicates that children with type 1 diabetes and celiac disease share three HLA class II loci in DRB3, DRB4 and DRB5 genes. *HLA.* (2021) 97:44–51. 10.1111/tan.14105 33043613 PMC7756432

[2] Gheshlagh RG Rezaei H Goli M Ausili D Dalvand S Ghafouri H Prevalence of celiac disease in Iranian patients with type 1 diabetes: a systematic review and meta-analysis. *Indian J Gastroenterol.* (2020) 39:419–25. 10.1007/s12664-020-01046-7 33263176

[3] Siddiqui K Nawaz SS Sumri NHA Alnaqeb D AlQurashi A Mujammami M. Celiac autoantibody positivity in relation to clinical characteristics in children with type 1 diabetes. *J Clin Transl Res.* (2020) 5:91–5. 32617423 PMC7326273

[4] Taczanowska A Schwandt A Amed S Toth-Heyn P Kanaka-Gantenbein C Volsky SK Celiac disease in children with type 1 diabetes varies around the world: an international, cross-sectional study of 57 375 patients from the SWEET registry. *J Diabetes.* (2021) 13:448–57. 10.1111/1753-0407.13126 33118261

[5] Zhao Z Zou J Zhao L Cheng Y Cai H Li M Celiac disease autoimmunity in patients with autoimmune diabetes and thyroid disease among chinese population. *PLoS One.* (2016) 11:e0157510. 10.1371/journal.pone.0157510 27427767 PMC4948776

[6] Zhou JMM Xin KJ Liu Y. Diagnostic value of tissue transglutaminase antibody and clinical characteristics analyze in type 1 diabetic patients with celiac disease (In Chinese). *Chin J Diabetes Mellitus.* (2017) 9:622–6.

[7] Chen LL Yang L. Regulation of circRNA biogenesis. *RNA Biol.* (2015) 12:381–8. 10.1080/15476286.2015.1020271 25746834 PMC4615371

[8] Haque S Harries LW. Circular RNAs (circRNAs) in health and disease. *Genes (Basel).* (2017) 8:12. 10.3390/genes8120353 29182528 PMC5748671

[9] Dube U Del-Aguila JL Li Z Budde JP Jiang S Hsu S An atlas of cortical circular RNA expression in Alzheimer disease brains demonstrates clinical and pathological associations. *Nat Neurosci.* (2019) 22:1903–12. 10.1038/s41593-019-0501-5 31591557 PMC6858549

[10] Zhang MY Wang JB Zhu ZW Li LJ Liu RS Yang XK Differentially expressed circular RNAs in systemic lupus erythematosus and their clinical significance. *Biomed Pharmacother.* (2018) 107:1720–7. 10.1016/j.biopha.2018.08.161 30257390

[11] Wang Z Huang K Xu J Liu J Zheng Y. Insights from dysregulated mRNA expression profile of beta-cells in response to proinflammatory cytokines. *J Immunol Res.* (2022) 2022:4542487. 10.1155/2022/4542487 35103245 PMC8800623

[12] Wang Z Deng C Zheng Y. Involvement of circRNAs in proinflammatory cytokines-mediated beta-cell dysfunction. *Mediators Inflamm.* (2021) 2021:5566453. 10.1155/2021/5566453 34054343 PMC8112919

[13] Mayer-Davis EJ Kahkoska AR Jefferies C Dabelea D Balde N Gong CX ISPAD clinical practice consensus guidelines 2018: definition, epidemiology, and classification of diabetes in children and adolescents. *Pediatr Diabetes.* (2018) 19:7–19. 10.1111/pedi.12773 30226024 PMC7521365

[14] Jalilian M Jalali R. Prevalence of celiac disease in children with type 1 diabetes: a review. *Diabetes Metab Syndr.* (2021) 15:969–74. 10.1016/j.dsx.2021.04.023 33946030

[15] Zhou WY Liu XY Wang MM Liang LP Liu L Zheng K Prevalence of celiac disease in China: meta-analysis and serological survey in high-risk populations. *J Dig Dis.* (2021) 22:645–55. 10.1111/1751-2980.13049 34482631

[16] Al-Hussaini A Sulaiman N Al-Zahrani M Alenizi A El Haj I. High prevalence of celiac disease among saudi children with type 1 diabetes: a prospective cross-sectional study. *BMC Gastroenterol.* (2012) 12:180. 10.1186/1471-230X-12-180 23259699 PMC3543703

[17] Maheshwari A He Z Weidner MN Lin P Bober R Del Rosario FJ. Influence of age and type 1 diabetes mellitus on serological test for celiac disease in children. *Pediatr Gastroenterol Hepatol Nutr.* (2021) 24:218–29. 10.5223/pghn.2021.24.2.218 33833977 PMC8007846

[18] Lionetti E Catassi C. Co-localization of gluten consumption and HLA-DQ2 and -DQ8 genotypes, a clue to the history of celiac disease. *Dig Liver Dis.* (2014) 46:1057–63. 10.1016/j.dld.2014.08.002 25200477

[19] Siddiqui K Uqaili AA Rafiq M Bhutto MA. Human leukocyte antigen (HLA)-DQ2 and -DQ8 haplotypes in celiac, celiac with type 1 diabetic, and celiac suspected pediatric cases. *Medicine (Baltimore).* (2021) 100:e24954. 10.1097/MD.0000000000024954 33725967 PMC7982179

[20] Yuan J Gao J Li X Liu F Wijmenga C Chen H The tip of the “celiac iceberg” in China: a systematic review and meta-analysis. *PLoS One.* (2013) 8:e81151. 10.1371/journal.pone.0081151 24324669 PMC3852028

[21] Goodwin G. Type 1 diabetes mellitus and celiac disease: distinct autoimmune disorders that share common pathogenic mechanisms. *Horm Res Paediatr.* (2019) 92:285–92. 10.1159/000503142 31593953

[22] Aljulifi MZ Mahzari M Alkhalifa L Hassan E Alshahrani AM Alotay AA. The prevalence of celiac disease in saudi patients with type 1 diabetes mellitus. *Ann Saudi Med.* (2021) 41:71–7. 10.5144/0256-4947.2021.71 33818147 PMC8020650

[23] Unal E Demiral M Baysal B Agin M Devecioglu EG Demirbilek H Frequency of celiac disease and spontaneous normalization rate of celiac serology in children and adolescent patients with type 1 diabetes. *J Clin Res Pediatr Endocrinol.* (2021) 13:72–9. 10.4274/jcrpe.galenos.2020.2020.0108 32820875 PMC7947719

[24] Svensson J Sildorf SM Pipper CB Kyvsgaard JN Bojstrup J Pociot FM Potential beneficial effects of a gluten-free diet in newly diagnosed children with type 1 diabetes: a pilot study. *Springerplus.* (2016) 5:994. 10.1186/s40064-016-2641-3 27398272 PMC4936999

[25] Creanza A Lupoli R Lembo E Tecce N Della Pepa G Lombardi G Glycemic control and microvascular complications in adults with type 1 diabetes and long-lasting treated celiac disease: a case-control study. *Diabetes Res Clin Pract.* (2018) 143:282–7. 10.1016/j.diabres.2018.07.031 30075178

[26] Imam S Dar P Aziz SW Zahid ZA Sarwar H Karim T Immune cell plasticity allows for resetting of phenotype from effector to regulator with combined inhibition of notch/eif5a pathways. *Front Cell Dev Biol.* (2021) 9:777805. 10.3389/fcell.2021.777805 34881246 PMC8645838

[27] Tompa A Akesson K Karlsson S Faresjo M. Suppressed immune profile in children with combined type 1 diabetes and celiac disease. *Clin Exp Immunol.* (2020) 201:244–57. 10.1111/cei.13454 32415995 PMC7419926

[28] Sheppard AL Elwenspoek MMC Scott LJ Corfield V Everitt H Gillett PM Systematic review with meta-analysis: the accuracy of serological tests to support the diagnosis of coeliac disease. *Aliment Pharmacol Ther.* (2022) 55:514–27. 10.1111/apt.16729 35043426 PMC9305515

[29] Carmona G Perera U Gillett C Naba A Law AL Sharma VP Lamellipodin promotes invasive 3D cancer cell migration via regulated interactions with Ena/VASP and SCAR/WAVE. *Oncogene.* (2016) 35:5155–69. 10.1038/onc.2016.47 26996666 PMC5031503

[30] Krause M Leslie JD Stewart M Lafuente EM Valderrama F Jagannathan R Lamellipodin, an Ena/VASP ligand, is implicated in the regulation of lamellipodial dynamics. *Dev Cell.* (2004) 7:571–83. 10.1016/j.devcel.2004.07.024 15469845

[31] Kurozumi S Joseph C Sonbul S Aleskandarany MA Pigera M Alsaleem M Clinicopathological and prognostic significance of ras association and pleckstrin homology domains 1 (RAPH1) in breast cancer. *Breast Cancer Res Treat.* (2018) 172:61–8. 10.1007/s10549-018-4891-y 30056565

[32] Hu X Jalal S Sheetz M Bakke O Margadant F. Correction: micro-stepping extended focus reduces photobleaching and preserves structured illumination super-resolution features. *J Cell Sci.* (2021) 134:258884. 10.1242/jcs.258884 34169963

[33] Redei EE Ciolino JD Wert SL Yang A Kim S Clark C Pilot validation of blood-based biomarkers during pregnancy and postpartum in women with prior or current depression. *Trans Psychiatry.* (2021) 11:68. 10.1038/s41398-020-01188-4 33479202 PMC7820442

[34] Sun H Lagarrigue F Wang H Fan Z Lopez-Ramirez MA Chang JT Distinct integrin activation pathways for effector and regulatory T cell trafficking and function. *J Exp Med.* (2021) 218:1524. 10.1084/jem.20201524 33104169 PMC7590511

[35] Husby S Koletzko S Korponay-Szabó IR Mearin ML Phillips A Shamir R European society for pediatric gastroenterology, hepatology, and nutrition guidelines for the diagnosis of coeliac disease. *J Pediatr Gastroenterol Nutr.* (2012) 54:136–60. 10.1097/MPG.0b013e31821a23d0 22197856

[36] Shahramian I Bazi A Sargazi A. An overview of celiac disease in childhood type 1 diabetes. *Int J Endocrinol Metab.* (2018) 16:e66801. 10.5812/ijem.66801 30214462 PMC6119207

