# Supplementary material for: Circulating circular RNA profiles associated with celiac disease seropositivity in children with type 1 diabetes
Source: Front Pediatr. 2022 Sep 23;10:960825. doi: 10.3389/fped.2022.960825 (PMC9537605; doi:10.3389/fped.2022.960825)
Supplement: Supplementary file 1 [file Table_1.DOCX]

**Supplementary Table 1** Primers used in target and reference genes amplification reactions

| Gene | Forward primer 5’→3’ | Reverse primer 3’→5’ |
| --- | --- | --- |
| *β-Actin* | GCAGATGTGGATCAGCAAGC | TTGTTTTCTGCGCAAGTTAGGT |
| *hsa_circ_0006561* | TGCGTTATCCTGTCAGCCAA | GGTTCAACTTCTACTCGTCGG |
| *hsa_circ_0004564* | GGCAGCCAATTACTGAGAGTTT | TGGCGAAGAGGTATATGTTGAAC |
| *hsa_circ_0041267* | TTCCTGCTTGCTGATTGCGT | TTGTTCTTACAGTGGTTACCGTG |
| *hsa_circ_0004712* | CCTCTGGACATCATGCTTGG | TATAGTCCATGTTGTATGAGTCCCC |
| *hsa_circ_0018827* | TGCTGCAGAGTGCTTTCTCTCT | CTAAGTTTGTCCGAAGAAGAACC |
| *RAPH1* | TCTTTGAGTATGGATGAGGCTG | GTGATGCTGGAATGGGAGG |

**Supplementary Table 2** The candidate circRNAs in T1D

| circRNAs | Log2(Fold change) | Change | Parental gene |
| --- | --- | --- | --- |
| *hsa_circ_0006561* | 1.136 | up | *KCNMA1* |
| *hsa_circ_0004564* | 1.070 | up | *RAPH1* |
| *hsa_circ_0041267* | 1.045 | up | *SLC43A2* |
| *hsa_circ_0004712* | -1.263 | down | *PDE7B* |
| *hsa_circ_0018827* | -1.100 | down | *USP54* |

**Supplementary Table 3**  Anthropometric features of the children with T1D with CDAb

|  | CDAb^+^(n=20) | CDAb^-^(n=27) | *p* value |
| --- | --- | --- | --- |
| Age at baseline (y) | 9.04±4.84 | 9.91±5.07 | 0.56† |
| Sex (M/F) | 11/9 | 12/15 | 0.47^¶^ |
| Duration of diabetes (mo) | 6.0(0.4,14.3) | 12.0(2.0,30.0) | 0.12* |

*Mann–Whitney U test, ^†^Student's t-test, ^¶^χ^2^ test, or Fisher's exact test. Data are presented as mean ±standard deviation or median (interquartile range: 25th–75th percentile).
